# UPR-Induced Resistance to Etoposide Is Downstream of PERK and Independent of Changes in Topoisomerase IIα Levels

**DOI:** 10.1371/journal.pone.0047931

**Published:** 2012-10-29

**Authors:** Melissa J. Mann, Ethel R. Pereira, Nan Liao, Linda M. Hendershot

**Affiliations:** 1 Department of Tumor Cell Biology, St. Jude Children's Research Hospital, Memphis, Tennessee, United States of America; 2 Department of Molecular Sciences, University of Tennessee Health Science Center, Memphis, Tenessee, United States of America; University of Hong Kong, Hong Kong

## Abstract

**Background:**

The unfolded protein response (UPR) is regulated by three ER-localized, transmembrane signal transducers that control distinct aspects of the UPR. We previously reported that both increased resistance to etoposide and a reduction in Topoisomerase IIα protein levels were a direct response of UPR activation, and the latter occurred independent of changes in Topo IIα mRNA levels. We have now examined the contribution of each of the three up-stream transducers of the UPR, as well as some of their downstream targets in affecting decreased expression of Topo IIα protein and increased drug resistance.

**Principal Findings:**

Our data revealed that while Ire1 activation led to Topo IIα loss at the protein level it did not contribute to changes in sensitivity to etoposide. The decreased expression of Topo IIα protein was not downstream of XBP-1, in keeping with the fact that Topo IIα transcription was not affected by ER stress. Conversely, PERK activation did not contribute to changes in Topo IIα protein levels, but it did play a significant role in the UPR-induced decreased sensitivity to etoposide. Several cellular responses downstream of PERK were examined for their potential to contribute to resistance. The ATF6 arm of the UPR did not significantly contribute to etoposide resistance within the time frame of our experiments.

**Conclusions and Significance:**

*In toto*, our data demonstrate that UPR-induced changes in Topo IIα protein levels are not responsible for resistance to etoposide as has been previously hypothesized, and instead demonstrate that the PERK branch plays a Topo IIα-independent role in altered sensitivity to this drug.

## Introduction

Once tumor cells have acquired the requisite genetic changes that lead to transformation, their microenvironment becomes the next hurdle to uncontrolled growth. After the tumor reaches a certain size with cells approximately ≥0.2 mm from a blood vessel [1],[2], they experience a decrease in the availability of nutrients that are required to support their rapid growth, insufficient levels of oxygen, and a build up of toxic, acidic waste products. This inadequate environment can cause the tumor cells to exit cell cycle and remain in a dormant state for many years [3]. Alternatively a number of stress-induced signal transduction pathways can be activated that allow the tumor cells to modify their environment and alleviate these problems, which contributes to the dysregulated growth associated with cancer.

The HIF (hypoxia inducible factor) pathway, which is induced by insufficient amounts of oxygen [4],[5],[6], is the best characterized stress pathway that is activated in rapidly growing tumor cells that have outgrown their vascular network [7],[8]. Under these conditions, the oxygen-labile α subunit of HIF is stabilized and forms a transcriptionally active complex with the more stable β subunit, which together regulates a large number of processes that are critical to tumor survival and growth, including the up-regulation of proangiogenic factors like VEGF [7]. More recently, it has been shown that decreased nutrients, particularly glucose, extreme hypoxia, and the build up of toxic waste products that result from inadequate vascularization can impinge on the normal homeostasis of the endoplasmic reticulum (ER) and negatively impact protein folding and assembly in this organelle [9],[10], [11]. This in turn induces the unfolded protein response (UPR), which largely serves to restore normal ER function, but under extreme or prolonged stress it can signal activation of proapoptotic pathways [12]. Indeed, a growing body of literature reveals that the UPR is activated in many different types of cancer [13],[14],[15]. Recent reports reveal that VEGF and a number of other proangiogenic factors are direct targets of the UPR [16],[17], which would be expected to promote angiogenesis and help mitigate the inadequate environment and allow the tumor to grow. In support of this possibility, inactivation of UPR signaling in various tumor models results in dramatically reduced tumor growth [18],[11],[19].

The UPR is signaled by three transmembrane proteins with luminal domains that sense the changes in the ER environment and cytosolically disposed effector domains. Ire1 was the first UPR transducer to be discovered in mammalian cells [20],[21] and is the homologue of the sole yeast UPR transducer [22],[23]. Mammalian cells possess two isoforms of Ire1; the ubiquitously expressed Ire1 α [20] and Ire1 β, which is expressed primarily in the stomach [21]. In addition to having a cytosolic kinase domain, the Ire1 proteins possess an endonuclease activity that is dependent on activation of its kinase domain [24]. The primary target of this activity in mammalian cells is the X-box binding protein-1 (XBP-1) transcript [25],[26],[27]. Upon activation, Ire1 excises 26 bases from *XBP-1* mRNA, which is then religated by an as yet undiscovered ligase. The removal of these bases changes the reading frame of the 3′ end of the message and transforms the spliced form of XBP-1 (XBP-1(S)) from a DNA binding protein that lacks a transactivation domain into a fully active transcription factor, which regulates a number of downstream components of the UPR [28]. In addition to XBP-1 transcripts, recent data suggest that Ire1 can cleave a number of mRNAs that are being translated on membrane bound polysomes [29], [30] and even ribosomes themselves [31], which helps to diminish the synthesis of secretory pathway proteins.

The second UPR transducer to be discovered is the PKR-like ER kinase (PERK), which is a member of the eIF-2α kinase family and serves to induce a transient inhibition of protein synthesis [32]. This helps to alleviate the further accumulation of unfolded proteins in the ER, but the block in protein synthesis is not restricted to ER proteins. Cyclin D1 mRNA has been shown to remain untranslated even after most protein synthesis is restored [33],[34], and the loss of this short lived protein is responsible for the G1 arrest that is associated with UPR activation [35],[36]. A second consequence of PERK activation and eIF-2α phosphorylation is the paradoxical translation of ATF4, which is poorly translated under non-stress conditions due to a series of small open reading frames in the 5′ region of the transcript that interfere with correct translation initiation [37]. Because up-regulation of ATF4 is shared with other stress-regulated eIF-2α kinases, this aspect of these pathways is referred to as the integrated stress response (ISR), where ATF4 has been shown to regulate a large number of elements critical to cell survival during a variety of stress conditions [38].

ATF6 is the third UPR transducer. Its N-terminal, cytosolically oriented domain encodes a transcription factor that remains tethered to ER membranes in the absence of stress due to a transmembrane domain that is followed by a C-terminal, luminal stress-sensing domain [39]. In response to UPR activation, ATF6 traffics to the Golgi where it is cleaved on both sides of its transmembrane domain by the S1P and S2P proteases, thus liberating the transcription factor domain [40]. In addition to the *XBP-1* gene, a number of ER chaperones and their co-factors are up-regulated by ATF6, which serves to prevent the aggregation of misfolded proteins and likely contributes to restoring ER homeostasis after the stress subsides [41]. The accumulation of misfolded or incompletely folded proteins in the ER serves as the signal for activating the UPR in all organisms studied from yeast to man [42]. This entails the release of BiP from the UPR transducers, which in the case of the kinases allows them to form higher order structures that activate in *trans* [43],[44], whereas the loss of BiP from the luminal domain of ATF6 is essential to allow ATF6 to be transported to the Golgi [45]. Data from yeast studies argue that the binding of unfolded proteins to the luminal domain of Ire1 can contribute to its oligomerization [46], [47]. However, crystallographic data on the mammalian orthologue suggest that the putative peptide binding cleft on Ire1 may not be large enough to accommodate a polypeptide chain [48], thus leaving this aspect of UPR activation in higher eukaryotes presently unresolved.

In addition to the UPR playing a critical role in tumor survival, many years ago pharmacological activation of the UPR in cultured cells was shown to alter their sensitivity to a number of chemotherapeutic agents [49],[50]. In the case of etoposide, UPR activation correlated with a decrease in expression of its target topoisomerase IIα [51], [52], providing a logical explanation for the significant increase in resistance to this drug. We previously reported that both the reduced expression of Topo IIα and the decreased sensitivity to etoposide were a direct effect of UPR activation [53]. This led us to posit that it should be possible to identify the responsible arm of the UPR and to ultimately target it to increase the sensitivity of tumors that showed evidence of UPR activation to this class of drugs. We report here that PERK was responsible for the decreased sensitivity to etoposide, but surprisingly PERK did not contribute to Topo IIα protein loss, which appeared to be downstream of Ire1 activation. However UPR-activated Ire1 null cells remained as resistant to etoposide as their wild-type counterparts, arguing that changes in Topo IIα expression and altered sensitivity to etoposide are two distinct and unrelated aspects of the mammalian UPR.

## Results

### Ire1 activation controls down-regulation of Topo II in an XBP-1(S)-independent manner

**Figure 1:**
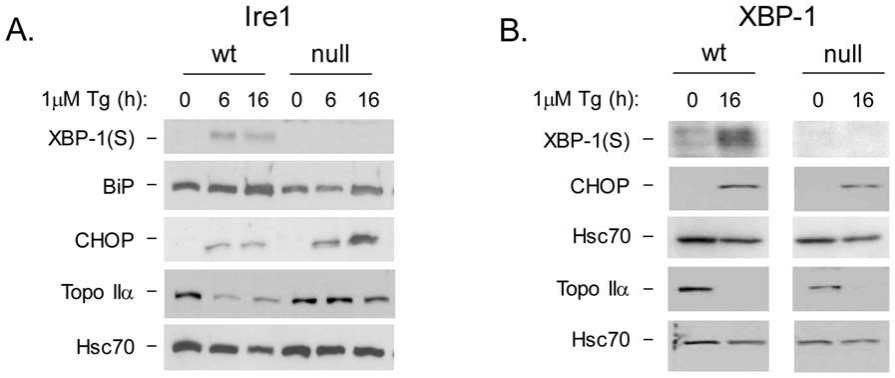
Ire1 regulates Topo IIα protein levels in an XBP-1 independent manner. Ire1 (**A**) or XBP-1 (**B**) wild-type and knock-out cells were treated with thapsigargin for the indicated times. Cell lysates were prepared and analyzed by western blotting with the indicated antiserum. Hsc70 serves as a control for lysate loading. Two gels were used in panel B; a 10% gel to separate the smaller XBP-1(S) and CHOP proteins and an 8% gel for the larger Topo IIα protein and thus there are two sets of loading controls.

An initial understanding of how ER stress might lead to decreased expression of Topo IIα came from a study that identified a stress-activated degron on this protein, which was targeted by Jab1/CSN5 to promote its degradation [54]. Jab1 was independently identified by another group as a protein that interacted with Ire1 in the absence of ER stress [55], suggesting that its release upon UPR activation might allow it to traffic to the nucleus to destabilize Topo IIα. Thus to begin our analysis, we examined Ire1 wild-type and null mouse embryonic fibroblast cells for UPR induced changes in Topo IIα expression. Indeed, we found that Ire1 null cells showed less degradation of Topo IIα than wild-type cells when treated with thapsigargin (Fig. 1A), although the level of stabilization in both types of cells varied considerably from experiment to experiment. As expected, loss of Ire1 resulted in the inhibition of XBP-1 splicing and only modestly affected BiP expression [26],[27]. In keeping with previous reports, the expression of CHOP, which is coordinately regulated by both PERK and ATF6, was somewhat increased suggesting there may be some compensation by other branches when one arm is disabled [56]. Ire1 has a kinase-dependent, endonuclease activity and the target of its endonuclease activity is the *XBP-1* mRNA. To determine if loss of Topo IIα was downstream of XBP-1, we next examined Topo IIα loss in XBP-1 wild-type and null cells after thapsigargin treatment. We found that UPR activation in both XBP-1 null cells and wild-type cells led to a similar decrease in Topo IIα expression, demonstrating that its loss was not downstream of XBP-1(S) (Fig. 1B), which is more consistent with the possibility that Ire1 directly regulates JAB1 localization. [54].

### Ire1 does not signal increased resistance to etoposide

**Figure 2:**
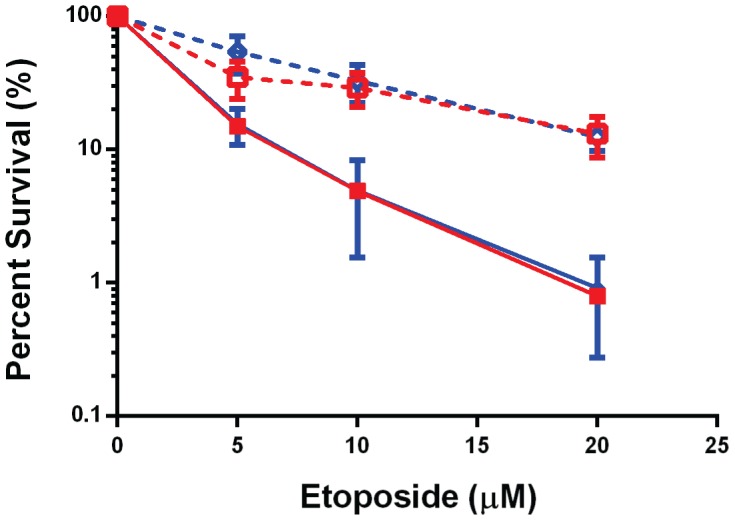
Ire1 does not contribute to increased resistance to etoposide in response to ER stress. Clonal survival assays were performed on Ire1 wild-type (blue) and knock-out (red) cells after 6 hrs of pretreatment with (dashed lines) or without (solid lines) thapsigargin follow by 2 hrs with the indicated concentrations of etoposide. The number of colonies formed for each concentration of etoposide is represented as a percent of those formed without etoposide. Data from three independent experiments are shown and the error bars indicate standard deviations.

**Figure 3:**
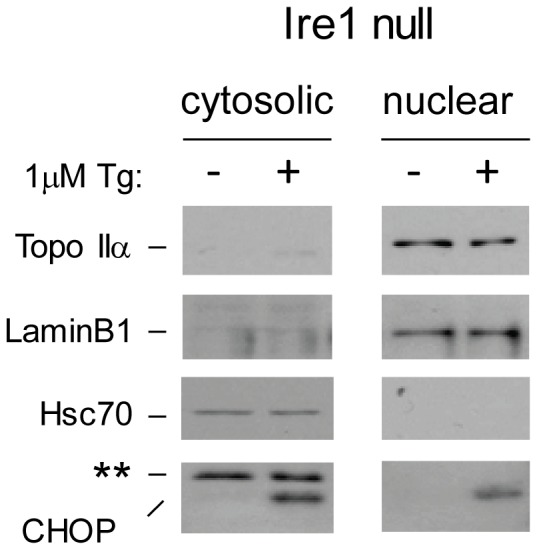
Decreased sensitivity to etoposide in Ire1 null cells that maintain Topo IIα expression is not linked to UPR induced changes in its localization. Ire1 knockout cells were treated with or without thapsigargin for 6 hrs and cells were homogenized and separated into a nuclear pellet and the cytosol. Fractions were subjected to western blotting with the indicated antibodies. LaminB1 serves as a control for the nucleus and Hsc70 for the cytosol. The asterisk indicates a non-specific band observed with the anti-CHOP antiserum.

To confirm that decreased expression of Topo IIα was responsible for the UPR-induced change in sensitivity of cells to etoposide, we examined the survival of wild-type and Ire1 null cells to etoposide before and after activation of the UPR. As anticipated, the wild-type and null cells were equally sensitive to etoposide before UPR activation. However, to our complete surprise ER stress led to a very similar increase in resistance to this drug in both lines (Figure 2), in spite of that fact that the levels of Topo IIα were decreased in the wild-type but not Ire1 null cells under these conditions. As this result was quite unexpected, we next determined if UPR activation might normally lead to changes in the nuclear expression of Topo II that might secondarily lead to its degradation. We reasoned that even though Topo II protein levels were not reduced in the Ire1 null cells, it might no longer be present in the nucleus where it would need to be for etoposide to exert its effects. Thus, we examined the subcellular localization of Topo II in the Ire1 null cells before and after thapsigargin treatment. As expected, Hsc 70 was restricted to the cytosol and laminB1 was found primarily in the nuclear fraction (Fig. 3), arguing that the fractionation was good. In keeping with a previous study, CHOP could be detected in both fractions [57]. When we next examined Topo IIα levels in the two fractions, we found that similar amounts of the protein were present in the nuclear fraction of Ire1 null cells both before and after ER stress (Figure 3). These results strongly argued that the correlation between stress-induced decreases in Topo IIα protein expression and the increased resistance to etoposide does not represent a causative relationship. Furthermore it argues that the reduced amount of Topo IIα protein remaining after UPR activation is still sufficient to serve as a target for etoposide-induced cell death.

### UPR-induced change in sensitivity to etoposide is downstream of PERK

**Figure 4:**
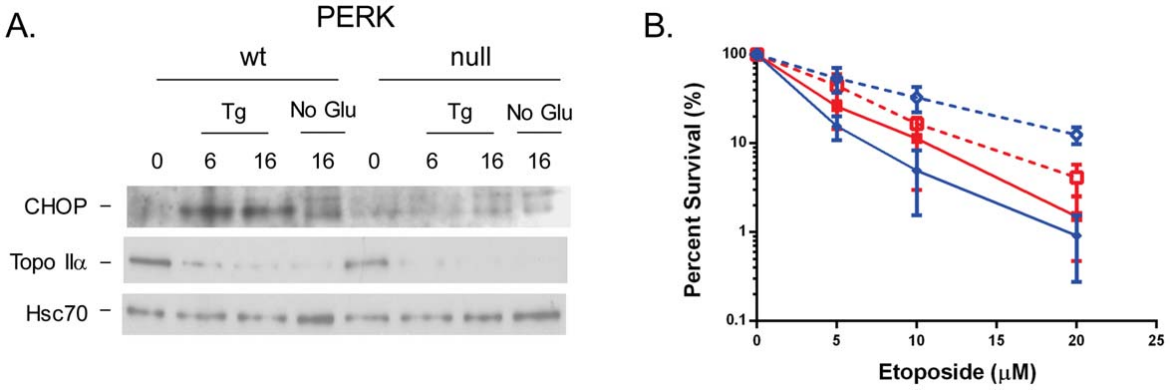
Altered sensitivity to etoposide is downstream of PERK and independent of changes in Topo IIα expression. **A.** PERK wild-type and knock-out cells were treated with thapsigargin for 0, 6 and 16 hrs or incubated in glucose-free media for 16 hrs. Cell lysates were prepared for western blotting as in Figure 1 and blotted with the indicated antisera. **B.** PERK wild-type (blue) and null (blue) cells were pretreated with (dotted lines) or without (solid lines) thapsigargin and then incubated with the indicated concentrations of etoposide. Clonal survival assays were performed as in Figure 2.

A second arm of the UPR is regulated by the PERK kinase, which phosphorylates eIF2α in response to ER stress, leading to a transient inhibition in protein translation. In experiments similar to those conducted on Ire1 wild-type and null cells, we first examined the expression of Topo IIα before and after UPR activation in PERK wild-type and null mouse embryonic fibroblast cells, although in this case we used both thapsigargin and no glucose as ER stress inducers. As a control, CHOP levels were examined and were not induced in the PERK null cells as expected (Figure 4A). In keeping with our conclusion that changes in Topo IIα levels were downstream of Ire1, we found that Topo II protein decreased similarly in both the PERK wild-type and null cells in response to both UPR inducers. We next examined the sensitivity of these cell lines to etoposide before and after UPR induction. Unlike the pair of Ire1 cells, which were indistinguishable from each other both before and after UPR activation, the PERK wild-type cells were slightly more sensitive to etoposide before ER stress than the null cells, but considerably more resistant after (Figure 4B). Treatment of the PERK null cells with thapsigargin provided a very modest increase in resistance to etoposide that was statistically significant at 5 and 20 µM but not at 10 µM, arguing that the majority of ER stress-induced resistance is downstream of PERK. This data further underscores the distinction between changes in Topo IIα protein levels and increased resistance to etoposide.

### PERK-induced cell cycle arrest is not responsible for UPR-induced resistance to etoposide

**Figure 5:**
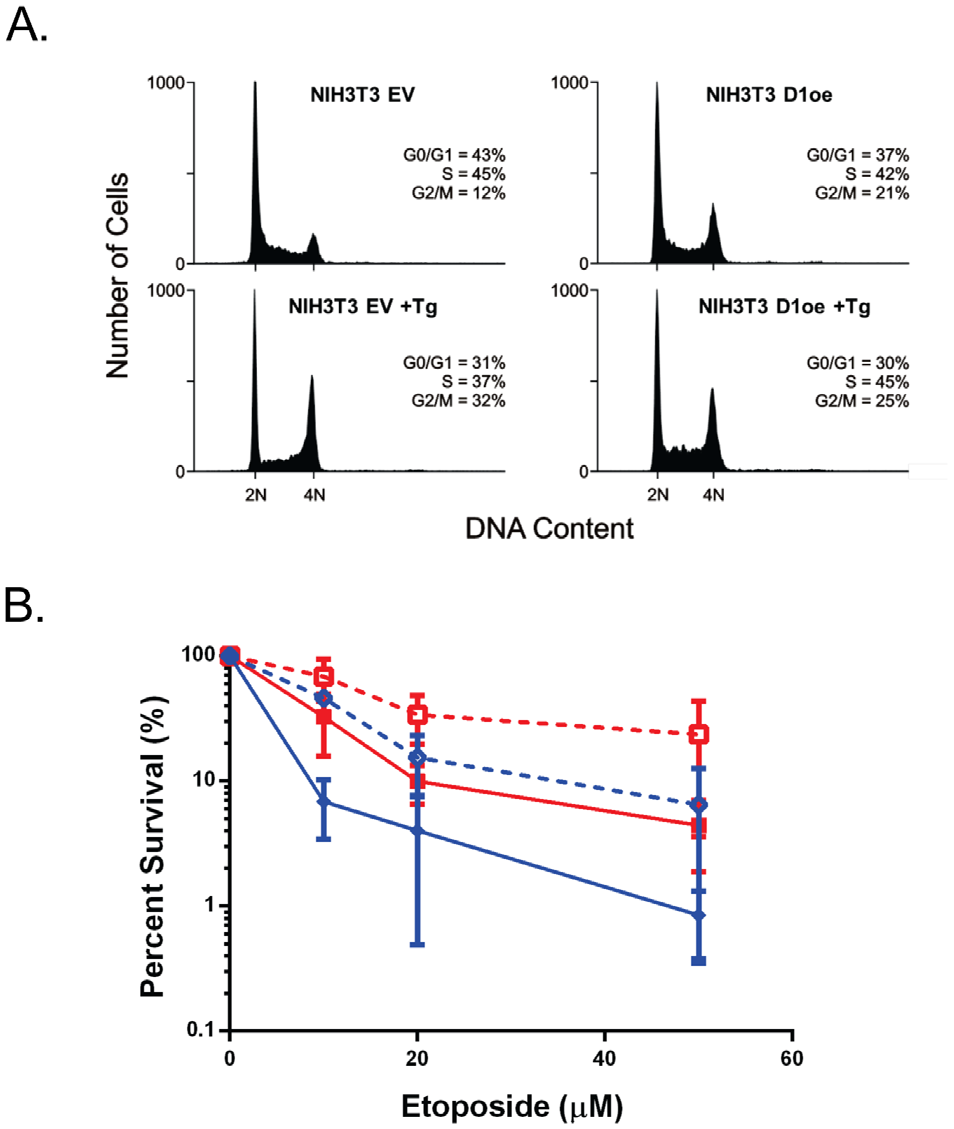
Decreased sensitivity to etoposide is not due to UPR-induced cell cycle arrest. **A.** NIH3T3 cells were transduced with a retrovirally transduced with an empty vector (ev) or a stable version of cyclin D1 (D1oe) and cell cycle analyses were performed after 0 and 6 h thapsigargin treatment. **B.** The same cells were plated for clonal survival assays.

The transient inhibition of translation that occurs downstream of PERK activation secondarily affects a number of cellular functions that could contribute to altered sensitivity to etoposide. Included among these is a G1 cell cycle arrest, which occurs due to the translational loss of cyclin D proteins [33],[34]. Since etoposide exerts its lethal effects by binding to replicating DNA when cells are in S phase, we wished to determine if UPR-induced changes in cell cycle were responsible for the altered sensitivity to etoposide. Importantly, G1 cell cycle arrest typically occurs after longer periods of UPR activation than we have used in our experiments [33], but it was conceivable that some changes in the percent of cells in S phase could be occurring by 6 hrs of UPR activation. To address this possibility, we used NIH 3T3 mouse fibroblasts that had been virally transduced with either an empty vector or with one encoding a more proteasome resistant cyclin D1 mutant [58], which allows cells to remain in cell cycle during prolonged ER stress [33]. Cell cycle analyses were performed on both cell lines in the absence of ER stress and after 6 hrs of thapsigargin treatment, which was the incubation time used for all of the clonal survival assays. Although the basal cell cycle distribution in the two lines is somewhat different, the percentage of cells in S phase is fairly similar; with the control line having ∼45% and the mutant D1 over-expresser having ∼42% (Figure 5A). After 6 hrs of thapsigargin treatment, the percent of cells in S phase in the control line had diminished modestly to ∼37%, whereas the D1 expressing cells remained at ∼45%. Next, the two lines were preincubated with or without thapsigargin for 6 hrs and then treated with etoposide, and clonal survival assays were conducted. Although the two lines showed distinct sensitivities to etoposide (the D1 over-expressers were actually more resistant), in both cases, activation of the ER stress response led to a decrease in sensitivity to etoposide with the D1 over-expressers becoming particularly resistant. The reason for the difference in basal sensitivity between the two lines is not clear, but these experiments clearly argue that ER stress-induced cell cycle arrest is not the cause of increased resistance to etoposide.

### ATF4 does not contribute to etoposide resistance

**Figure 6:**
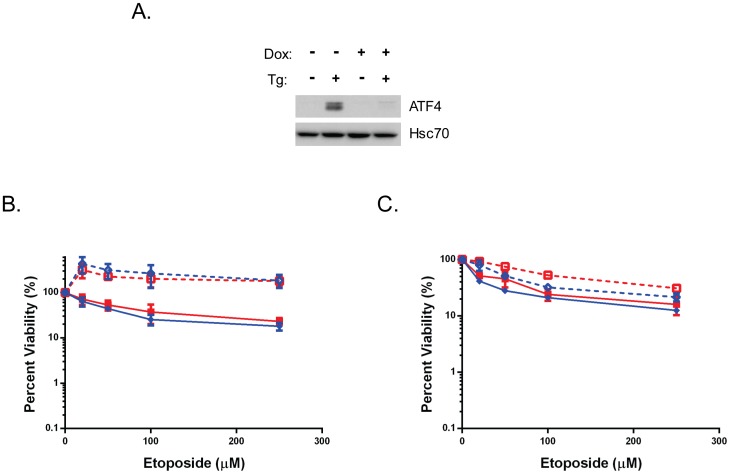
ATF4 does not contribute significantly to UPR-induced resistance to etoposide. **A.** The SK-N-AS clone expressing inducible shRNA specific for ATF4 was either left untreated (−) or treated with 1 µg/ml doxycyline (+) for 48 hours. The cells from each treatment were divided in half and either left untreated (−) or treated with thapsigargin (+) for 16 hours. Cells were harvested and ATF4 protein levels were measured using western blot analysis. Hsc70 serves as a control for loading. **B.** The ATF4shRNA clone was either left untreated (blue) or treated with doxycycline (1 µg/ml media, red) for 48 hours, after which the cells were either left untreated (solid lines) or treated with 1 µM thapsigargin for 6 hours (dotted lines), followed by the indicated amounts of etoposide for 2 hours as indicated. Data from three independent experiments are shown and the error bars indicate standard deviations. **C**. The ATF4shRNA clone was treated as in B, except that only 0.06 µM thapsigargin was used to induce the UPR. Cell viability in both cases was determined using the Pierce CellTiter assay as described in the Materials and Methods section.

The ATF4 transcription factor is downstream of PERK activation in response to ER stress but can also be induced by a number of other stress pathways, as well as alterations in basal levels of eIF2α phosphorylation [59]. Basal levels of ATF4 have been shown to be increased in some human tumor lines [60], [61], [62], [63], although the pathway that is responsible for its expression in these lines was not determined. Importantly, knocking down ATF4 expression in some of these lines increased their sensitivity to cisplatin [60], [61] Bortezomib [62] and a number of other agents including etoposide [63] under normal cell culture conditions. Thus, we next explored the contribution of this protein to UPR-induced resistance to etoposide. First we examined the effects of UPR activation on etoposide sensitivity in wild-type and ATF4 null primary MEFs. The cells are not transformed and grow very slow with low numbers of cells in S or G2 phase [64], which resulted in us having to use much higher concentrations of etoposide. In the case of WT cells, activation of the UPR resulted in a very modest increase in protection (7% versus 19% surviving with 250 µM etoposide), whereas UPR activation in the ATF4 null cells actually did result in a significant increase in resistance (8% versus 41% surviving with 250 µM etoposide) (Figure S1). This suggested that ATF4 was not critical to protection but given the low sensitivity to etoposide and the modest increase in UPR-mediated protection in these cells, we were reluctant to draw this conclusion. Thus, we took another approach, which was to engineer the SK-N-AS human neuroblastoma cell line with inducible expression of shRNA to ATF4. We were able to achieve very good inhibition of ATF4 expression in response to ER stress (Figure 6A). When these cells were subjected to viability assays, we found that there was no difference in increased resistance to etoposide after thapsigargin treatment whether or not ATF4 was expressed. However, the SK-N-AS cells were quite sensitive to the usual concentration of thapsigargin and actually survived better when it was combined with etoposide (Figure 6B). We do not understand the reason for this, so we titered thapsigargin down to a concentration in which these cells survived and still showed evidence of UPR activation (Figure S2). At 0.06 µM thapsigargin (1/16 the usual concentration, the UPR-induced protection was less robust but was very similar in both the control (without Dox) and ATF4 knockdown (with Dox) cells (Figure 6C). Together these data suggest that ATF4 is not playing a significant role in etoposide resistance during ER stress conditions.

### ATF6 does not appear to contribute significantly to etoposide resistance

There was a small, but detectable change in resistance to etoposide in the PERK null cells after treatment with thapsigargin, which was significant at both 5 and 20 µM etoposide. Thus we wished to determine if the third UPR transducer ATF6 also provided some protection, since previously published data found that over-expression of BiP, which is downstream of this arm of the UPR protected against etoposide [65]. We obtained transformed ATF6 wild-type and null cells for these experiments. Unfortunately, as previously reported, the null cells were quite sensitive to standard concentrations of ER stress inducing agents [41]. In hopes of alleviating this problem, we titered tunicamycin and thapsigargin to obtain the lowest concentration of either agent that would induce a UPR. We found that 0.09 µg/ml of tunicamycin (versus 2.5 µg/ml in standard assays) was sufficient in both lines to activate the UPR within 3 hrs as indicated by CHOP and Herp expression. However, while the wild-type MEFs were ∼100% viable after this treatment, more than 30% of the ATF6 null MEFs were killed (data not shown), making it impossible to examine any potential contribution of this arm of the UPR to etoposide resistance using these cells.

**Figure 7:**
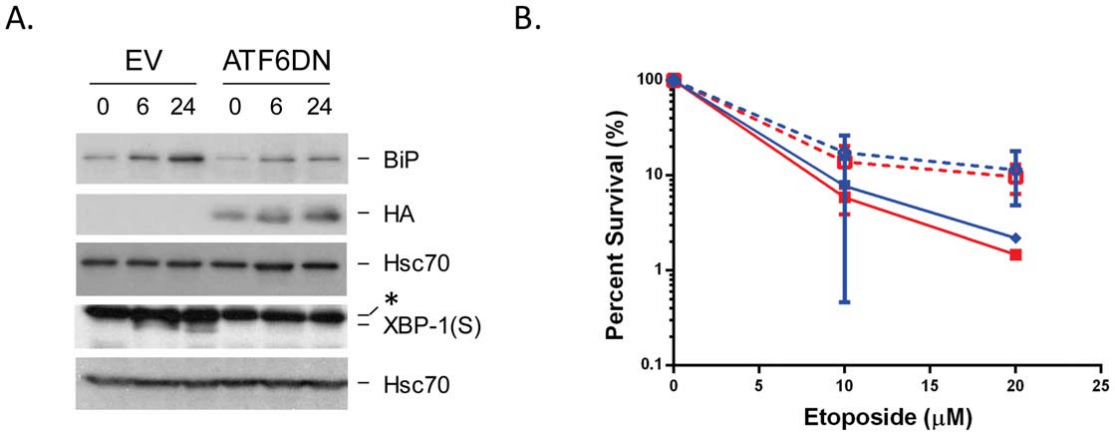
ATF6 does not appear to contribute significantly to altered etoposide sensitivity. **A.** 293T cells were transfected with empty vector or a dominant negative form of ATF6. 24 hrs later they were treated with thapsigargin for 6 and 24 hrs and cell lysates were prepared, separated on SDS gels, transferred and blotted for the indicated proteins. The asterisk represents a non-specific band recognized by the anti-XBP-1 antiserum. **B.** Wild-type MEFs were co- transfected with GFP and either an empty vector (blue) or a dominant negative form of ATF6 (red) as described in the Materials and Methods section. GFP-positive cells were isolated and either left untreated (solid lines) or pre-treated with thapsigargin for 6 hrs (dotted lines) before incubating with the indicated concentrations of etoposide. Clonal survival assays were performed as in Figure 2.

As an alternative approach, we engineered a dominant negative construct of ATF6 that contained the DNA binding domain without a transactivation domain and found that it inhibited the up-regulation of BiP and XBP-1(S) two targets of ATF6 (Figure 7A and Figure S3). This construct or an empty vector was co-transfected with a GFP construct into the PERK wild-type MEFs, which showed easily measurable UPR-induced resistance to etoposide (Figure 4B). Twenty four hrs after transfection, GFP^+^ cells were sorted and allowed to recover overnight. To determine whether the dominant negative construct was able to efficiently block this arm of the UPR, we first pre-incubated cells with or without thapsigargin for 6 hrs and then with etoposide for 2 hrs as done in the previous experiments. The four groups of cells were next subjected to clonal survival assays. The expression of GFP and sorting did not appear to affect the etoposide sensitivity of the PERK wild-type cells that did not express the dominant negative ATF6 construct either before or after thapsigargin treatment (compare Figures 4B and 7B). When cells expressing the mutant ATF6 were similarly examined, we found that there was no evidence of a decrease in the protection to etoposide afforded by the UPR, thus arguing that within the time frame of these experiments ATF6, or its downstream targets, are not contributing to resistance.

## Discussion

A number of previous studies have demonstrated that induction of the ER stress response leads to a decrease in Topo IIα protein [51],[53],[52] and a concomitant resistance to chemotherapeutic agents that target this enzyme [49],[50] leading investigators, including ourselves, to suggest a causative link. Topo IIα binds to DNA and forms double strand breaks that allow the DNA to unwind during replication [66],[67]. Drugs that target this enzyme stabilize the breaks and activate DNA damage checkpoints, which result in cell death [68]. It has been hypothesized that the reduced amount of Topo IIα found during ER stress results in fewer double strand breaks and therefore less cell death. However, the data presented in this study clearly separate these two events. Using Ire1 null and wild-type matched MEFs, we found that the loss of Topo IIα was downstream of Ire1. However Ire1 null MEFs, which did not have a significant UPR-induced decrease in Topo IIα protein and still localized this protein to the nucleus, were still more resistant to etoposide in response to ER stress. Thus although Topo IIα levels decrease when the UPR is activated, it appears that sufficient quantities remain to serve as a target for etoposide.

A previous study reported that prolonged ER stress led to a reduction in Topo IIα transcripts [50]. Thus, one mechanism by which Ire1 could contribute to the reduction of Topo IIα expression is through its endonuclease activity, since activated Ire1 was recently shown to digest a number of mRNAs [29],[30]. However, Ire1 appears to preferentially target those transcripts that are translated on ER membranes [29],[30]. Also, Topo IIα mRNA is not affected by the shorter incubations with UPR inducers that are sufficient to protect cells from etoposide and to result in a decrease in Topo IIα protein expression [50],[53]. This argues that Ire1-dependent loss of Topo IIα is not due to its mRNA being a direct target of Ire1's endonuclease activity. Instead, our data are more compatible with two previous reports. In one case, glucose deprivation induced the proteasomal degradation of Topo IIα protein, which was dependent on Jun activating binding protein-1 (JAB1) [54], and in a separate study, Jab1 was found to bind to Ire1α in the absence of ER stress [55]. Together the data from these studies are compatible with a model in which activation of Ire1 results in the release of Jab1 and its resultant trafficking to the nucleus where it acts to destabilize Topo IIα protein, although this has not yet been demonstrated. However, since decreasing the expression of Topo IIα does not appear to contribute significantly to etoposide resistance during ER stress, we chose not to formally test this model.

A second mechanism that has been proposed for UPR-induced resistance to chemotherapeutic agents is the increased expression of the ER chaperone BiP. One study compared the Chinese hamster ovary CHO cell line to one that stably over-expressed BiP and found that the BiP over-expressing line was significantly more resistant to etoposide, doxorubicin and camptothecin in the absence of treating with UPR inducers [65]. The BiP over-expressing cells had a portion of BiP in the cytosol where it prevented the activation of caspase-7 and blocked apoptosis. However, in the time frame of our experiments, we did not detect significant induction of BiP expression. Another study examined two paired cell lines that show characteristics of dormancy versus rapid tumor formation in mice. The dormant cell line was more resistant to etoposide and doxorubicin in culture, and this was linked to both constitutive PERK activation and higher basal levels of BiP in this line [69]. Blocking BiP expression with siRNA or PERK activity with dominant negative mutants both led to increased sensitivity of the dormant line to these drugs, although it is unclear if each of these components of the UPR acted separately or if one or the other of these approaches affected the other arm. Another study found that cytosolic extracts produced from cells that had been treated with UPR-inducers for 24 hrs had an activity that blocked caspase activation by cytochrome c, and this activity was lost when BiP was immunodepleted from the cytosolic extract [70]. However, some caution is warranted in interpreting these data. BiP is a soluble ER protein and can readily leak from ER vesicles during their preparation, and it is not entirely clear that the levels of BiP normally achieved by UPR activation are sufficient to provide a significant cytosolic pool of this protein in live cells to block apoptosis. In fact, apoptosis via caspase activation is a normal consequence of prolonged ER stress in many types of cells [71]. Lastly, the resistance of tumor-associated endothelial cells to chemotherapeutic agents has been linked to high BiP levels, and reducing these with siRNA increased their sensitivity [72]. Thus, several studies have found that high levels of BiP can increase resistance to etoposide, either through enhancing the proteostasis of the cell or inhibiting the cleavage of caspases, and argue that this arm of the UPR may provide a second measure of protection with prolonged stress.

Our study revealed that activation of the PERK branch of the UPR was the major contributor to UPR-induced etoposide resistance, which was independent of changes in Topo IIα protein expression. There are a number of PERK targets that could provide cellular protection to etoposide. The first of these is a PERK-mediated G0/G1 cell cycle arrest that occurs due to loss of the short lived cyclin D1 protein [34]. This could clearly provide protection, since Topo IIα targeted therapies work in S phase when DNA is being replicated, and cells with a chronically activated UPR often have many fewer cells in cycle. If this was the cause of increased resistance, we reasoned that it was likely to be a cell culture artifact that would be unlikely to apply to tumor cells growing *in vivo*, as they often have a genetically dysregulated cell cycle that provides them with unchecked growth. To rule this possibility out, we first examined cell cycle distribution in our cells before and after thapsigargin/etoposide treatment. We found that the short treatment time did not significantly alter cell cycle distribution. Nonetheless, we used an NIH-3T3 line that over-expresses a mutant form of cyclin D1, which is much more stable and renders cells resistant to UPR-induced cell cycle arrest [33]. These cells were actually slightly more resistant to etoposide than the parental line both before and after UPR activation. Together these data argue against protection occurring due to the UPR reducing the number of cells in S phase.

A second PERK-mediated possibility is that protection is afforded by an as yet unidentified target of ATF4 and the integrated stress response, which is shared by a number of cellular stresses that activate various eIF2α kinases [37]. Downstream components of the ISR help promote survival in response to a number of cellular stresses including oxidative stress [38]. ER stress also protects cells against anthracyclines, like doxorubicin and daunorubicin [49]. In addition to targeting Topo IIα, this class of drugs generates free radicals that cause oxidative damage to the cell due to the addition of an electron to their quinone ring that is resolved by reducing oxygen to reactive oxygen species [73]. While the ISR might provide some protection to these agents, Topo IIα-targeting podophyllotoxins, like etoposide and teniposide, do not possess a quinone ring and thus do not cause oxidative damage to the cell. In fact recent studies have demonstrated that lowering ATF4 levels in several lines that had not been treated with ER stress inducing agents increased their sensitivity to a number of alkylating agents, Bortezomib, and even etoposide. However, when we examined the contribution of this transcription factor to UPR-induced protection using two cell lines and two approaches, we did not observe a significant role for ATF4 in resistance to etoposide. A third possibility is that although PERK is often considered to contribute to the cell death aspects of the UPR due to its downstream target CHOP [74], although in some cellular contexts CHOP has been shown to have prosurvival functions [75],[76]. Thus it is impossible to automatically eliminate this target of PERK. However, when we compared CHOP null and wild-type cells, they both had a similar increase in resistance to etoposide in response to ER stress (data not shown). Finally, activation of PERK, and the resulting eIF-2α dependent inhibition of translation, can lead to NF-κB activation through the translational suppression of the short-lived inhibitory kappa B (IκB) [77] and/or dissociation of this inhibitor [78]. In many cellular contexts, NF-κB promotes cell survival [79],[80] and has been linked to chemoresistance in response to other stimuli [81]. Further experiments are underway to determine if NF-κB or one of the other components downstream of the PERK pathway provides resistance to Topo IIα-targeted therapies in response to ER stress and the mechanism by which it does so.

In summary, these studies provide strong evidence that the UPR-induced decrease in Topo IIα protein expression is not responsible for the increased resistance to chemotherapeutic agents that target this enzyme. In addition, we demonstrate that protection afforded by more short term ER stress responses does not involve the ATF6 branch that leads to the up-regulation of BiP, although it is possible that this ER chaperone plays an additional protective role during chronic UPR activation. Finally we demonstrate that resistance to etoposide is downstream of PERK activation but independent of the G1 arrest associated with this arm of the UPR.

## Materials and Methods

### Ethics Statement

A mouse NIH3T3 fibroblast cell line that over-expresses cyclin D1 and control line were obtained from the lab of Dr. Charles Sherr. The original paper for producing these lines is cited. The Ire1 and PERK null mouse fibroblasts were produced in the lab of Dr. David Ron and the XBP-1 null mouse fibroblasts were produced in Dr. Laurie Glimcher's lab. The original manuscripts describing each of these knockout mice and the isolation of MEF lines is referenced.

### Cell lines

Wild-type, Ire1 null [82], and PERK null MEFs [83] were kind gifts from Dr. David Ron (Skirball Institute, NYU, New York, NY). Cells were cultured in DMEM supplemented with 10% fetal bovine serum, 2 mM glutamine, 10 µg/ml gentamicin, 1× non-essential amino acids, 55 µM 2-ME at 37°C in a 5% CO_2_ incubator. XBP-1 wild-type and null MEFs [28] were kindly provided by Dr. Laurie Glimcher (Harvard University). ATF4 wild-type and null primary MEFs were generated in our lab and described previously [64]. Cells were cultured in DMEM (Life Technologies catalog #11960) supplemented with 10% fetal bovine serum, 2 mM glutamine, 10 µg/ml gentamicin, 1× non-essential amino acids, 55 µM 2-ME at 37°C in an 8% CO_2_ incubator. NIH3T3 mouse fibroblast cells over-expressing a cyclin D1 mutant engineered to prevent cell cycle arrest (NIH3T3 D1^oe^) were described previously [33]. SK-N-AS cells were derived from a metastatic human neuroblastoma, which was adapted to tissue culture [84], and 293T cells are a human embryonic kidney line transformed with large T antigen. Cells were grown in DMEM supplemented with 10% fetal bovine serum, 2 mM glutamine and 1% antibiotic-antimycotic at 37°C in a 5% CO_2_ incubator.

### Western blotting

Cells were pelleted and stored at −80°C prior to lysis in SDS lysis buffer (50 mM Tris-HCl [pH 8.0], 0.6% SDS). Cell pellets were disrupted by pipetting, and samples were incubated on ice for approximately 30 minutes. Lysates were sonicated in 30 second pulses with 20 second breaks for 4–6 minutes, incubated at 95°C for 10 min, and diluted with a 4-fold volume of mild lysis buffer (10 mM sodium phosphate [pH 7.2], 2 mM EDTA, 0.25 M NaCl, and 0.1% NP-40). Lysates were centrifuged for 20 min at 15,000 rpm to remove debris. All lysis buffers contained 0.25 mM phenylmethylsulphonyl fluoride (PMSF) and a protease inhibitor cocktail (Complete, Roche). Antibodies specific for Hsc70, LaminB1, and the spliced form of XBP-1 were purchased from Santa Cruz Biotechnologies. Affinity purified rabbit anti-Topo IIα antiserum [53], polyclonal anti-CHOP [33], and polyclonal anti-rodent BiP [85] were produced in our laboratory as described.

### Survival assays

Survival in response to etoposide treatment was measured in one of two ways. In the case of clonal survival assays, cells were treated or left untreated for six hours with 1 µM thapsigargin (Tg), washed twice with PBS, and put in media containing the indicated concentrations of etoposide for 2 hours as described previously [53]. Following treatments, cells were harvested by trypsinization, plated in triplicate and allowed to grow until they formed colonies of approximately 20 cells. Colonies were detected by staining with crystal violet and quantified by dividing by the number of cells plated. The survival of non-treated cells was set to 100% and those treated with the various concentrations of etoposide were expressed as a percentage of the untreated cells. In the case of pre-incubation with thapsigargin, colony formation was set relative to thapsigargin treated cells that did not receive etoposide. Except in the case of ATF6 null MEFs and the SK-N-AS neuroblastoma line (see below), comparison of Tg-treated to Tg-untreated controls revealed that no more than 20% death was caused by Tg treatment alone. In each case, the experiment was repeated at least three times, and standard deviation was determined. Alternatively, a cell viability assay was used to measure survival after etoposide treatment in some cases. Cells were treated or left untreated for six hours with 1 µM thapsigargin (Tg), washed twice with PBS, and put in media containing the indicated concentrations of etoposide for 2 hours as described previously [53]. Following treatments, cells were harvested by trypsinization, plated in quadruplicate on 96 well dishes and allowed to grown for 6–8 days. Cell viability was measured using the “CellTiter 96R AQueous Non-Radioactive Cell Proliferation Assay” (Pierce catalog #G5430).

### Generation of ATF4 deficient neuroblastoma cell line using inducible shRNA expression

shRNA sequence targeting human ATF4 was cloned into the pSuperior-puro vector (Oligoengine), which expresses shRNA molecules from a doxycycline-inducible H1 promoter. Two separate vectors, one containing the tetracycline repressor and the other encoding the ATF4 shRNA were stably transfected in the SK-N-AS cell line. Blasticidin (3 µg/ml media) and puromycin (1 µg/ml media) were used to select for clones stably expressing the Tet-repressor and p-Superior vectors respectively. After obtaining antibiotic-resistant double transfectants, a number of individual clones were picked using cloning cylinders and screened for regulated knock-down of ATF4 expression. The ATF4 shRNA sequence used to clone in the pSuperior vector with *BglII* and *XhoI* overhangs is as follows:

forward 5′- gatccccccttctgaccacgttggatttcaagagaatccaacgtggtcagaaggttttta-3′ and reverse 5′- tcgataaaaaccttctgaccacgttggattctcttgaaatccaacgtggtcagaaggggg-3′

### ATF6 dominant negative studies

Full length ATF6 cDNA was a kind gift from Dr. Ron Prywes and was used to produce a dominant negative deletion mutant corresponding to amino acids 171–373, which encode the DNA binding domain, followed by an HA tag (pCGN-HA-ATF6 DN). Wild-type MEFs were transfected with GFP alone or co-transfected with GFP and pCGN-HA-ATF6 using GeneCellin (BioCellChallenge) according to the manufacture's recommendation. Twenty four hours post transfection cells were sorted for GFP and allowed to recover overnight. Clonogenic survival assays on sorted cells were performed as described above.

### Subcellular fractionation

Cells were lysed in Nonidet P-40 lysing buffer (50 mM Tris-HCl [pH 7.5], 150 mM NaCl, 0.5% Nonidet P-40, and 0.5% deoxycholic acid) for 30 minutes on ice. Lysates were centrifuged for 20 min at 15,000 rpm. The supernatant containing the cytosolic fraction was kept on ice, while the pellet was further incubated with SDS lysis buffer, sonicated in 30 second pulses with 20 second breaks for 4–6 minutes, boiled for 10–20 min, and diluted with 4-fold volume of mild lysis buffer. The resulting nuclear fraction was then centrifuged at 15,000× g for 10 min to remove debris. A Bradford assay was performed to quantify the protein in the various cytosolic fractions, which were normalized to each other. Then in each case, the same adjustment was made to the corresponding nuclear fractions, before loading onto gels.

### Cell cycle analysis

NIH3T3 and NIH3T3 cells over-expressing cyclin D1 were treated for six hours with 1 µM thapsigargin or left untreated. Following treatment cells were stained with propidium iodide and DNA content was analyzed by flow cytometry.

### RNA Isolation and two-step Real-Time PCR

Total RNA was isolated from cells using RNeasy Plus Kit from Qiagen following manufacturer's instructions. 2 µg of total RNA were converted to cDNA using the High-capacity cDNA reverse-transcription Kit from Applied Biosystems. Real-time PCR analysis was performed on the ABI PRISM 7500HT Sequence Detection System using the SYBR Green PCR master mix (ABI). The primer sequences are as follows: for GRP170 the forward primer was 5′-gtgctgcagctcatcaatgac-3′ and reverse was 5′-atctgcagctgtggctgcatc-3′, for BiP the forward primer was 5′- gttcttgccgttcaaggtgg-3′ and reverse was 5′-tggtacagtaacaactgcatg-3′, for β-Actin the forward primer was 5′-gagaccttcaacaccccagcc-3′ and the reverse was 5′-ggatcttcatgaggtagtcag-3′, and for XBP1 (total) the forward primer was 5′-ccaaggggaatgaagtgagg-3′reverse was 5′-aagttgtccagaatgcccaacag-3′ [86].

## Supporting Information

Figure S1 ATF4 wild-type (blue) and knock-out (red) MEFs were pretreated with (dashed lines) or without (solid lines) 0.1 µM thapsigargin for 6 hrs followed by 2 hours with the indicated concentrations of etoposide. Viability was determined using the Pierce CellTiter assay as described in materials and methods.
(TIF)

Figure S2 The non-doxycline treated SK-N-AS clone was treated with the indicated concentrations of thapsigargin for 6 hours. Cell lysates were prepared and analyzed by western blotting with the indicated antiserum. Hsc70 serves as a control for lysate loading. Cell viability was determined using the Pierce CellTiter assay as described in materials and methods and is indicated as % survival under each lane.
(TIF)

Figure S3 293T cells were transiently transfected with either pCDNA3- empty vector (black bars) or pCGN-ATF6- dominant negative vector (grey bars). After 24 hours the cells were treated with thapsigargin (1 µM) for either 6 hours or 24 hours as indicated in the figure. Total RNA from the indicated samples was extracted and subjected to qRT-PCR to quantify *BiP*, *XBP-1*, and *GRP170* mRNA levels. RNA levels were expressed relative to the control untreated samples transfected with each of the vectors, which was set to 1.
(TIF)
