# Supplementary figures and images for: UPR-Induced Resistance to Etoposide Is Downstream of PERK and Independent of Changes in Topoisomerase IIα Levels
Source: PLoS One. 2012 Oct 29;7(10):e47931. doi: 10.1371/journal.pone.0047931 (PMC3483293; doi:10.1371/journal.pone.0047931)

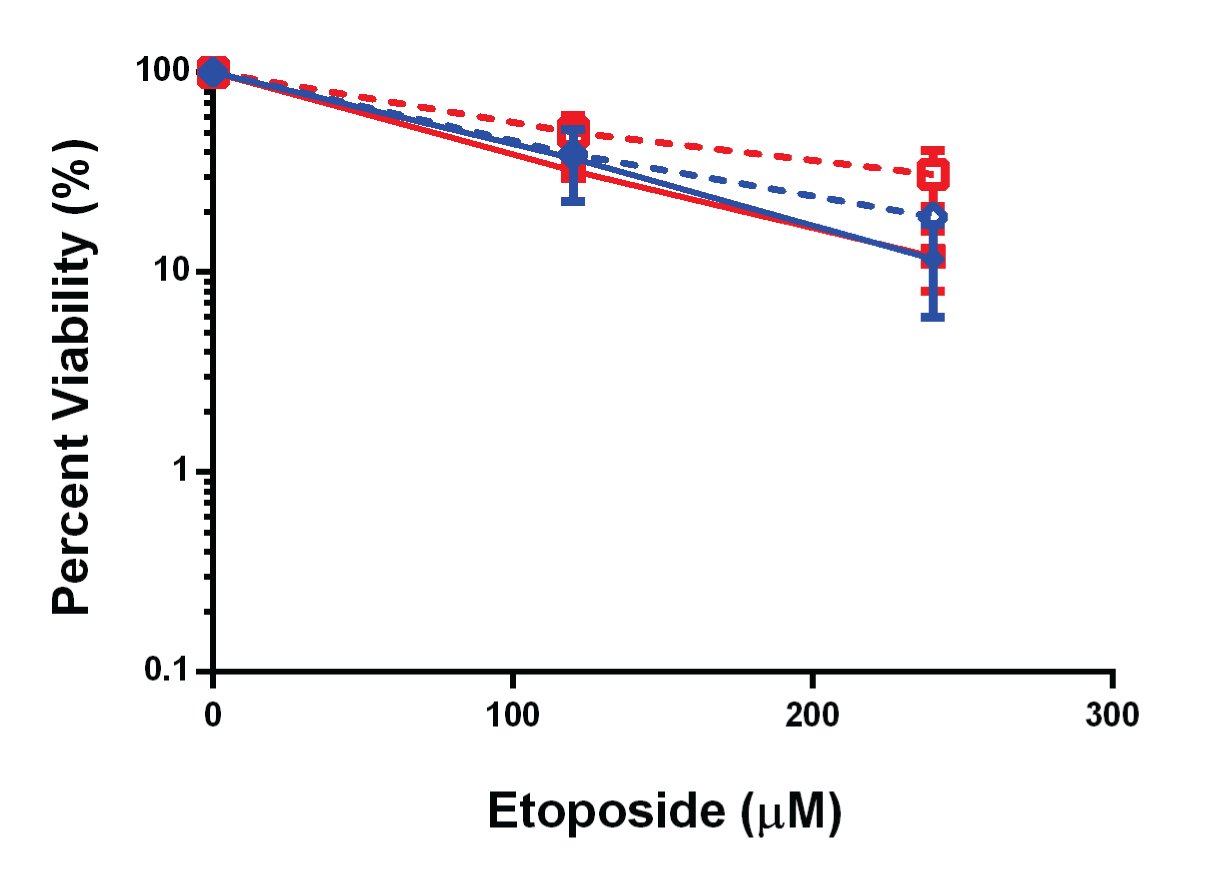

Supplement: Figure S1 — ATF4 wild-type (blue) and knock-out (red) MEFs were pretreated with (dashed lines) or without (solid lines) 0.1 µM thapsigargin for 6 hrs followed by 2 hours with the indicated concentrations of etoposide. Viability was determined using the Pierce CellTiter assay as described in materials and methods. (TIF) [file pone.0047931.s001.tif]

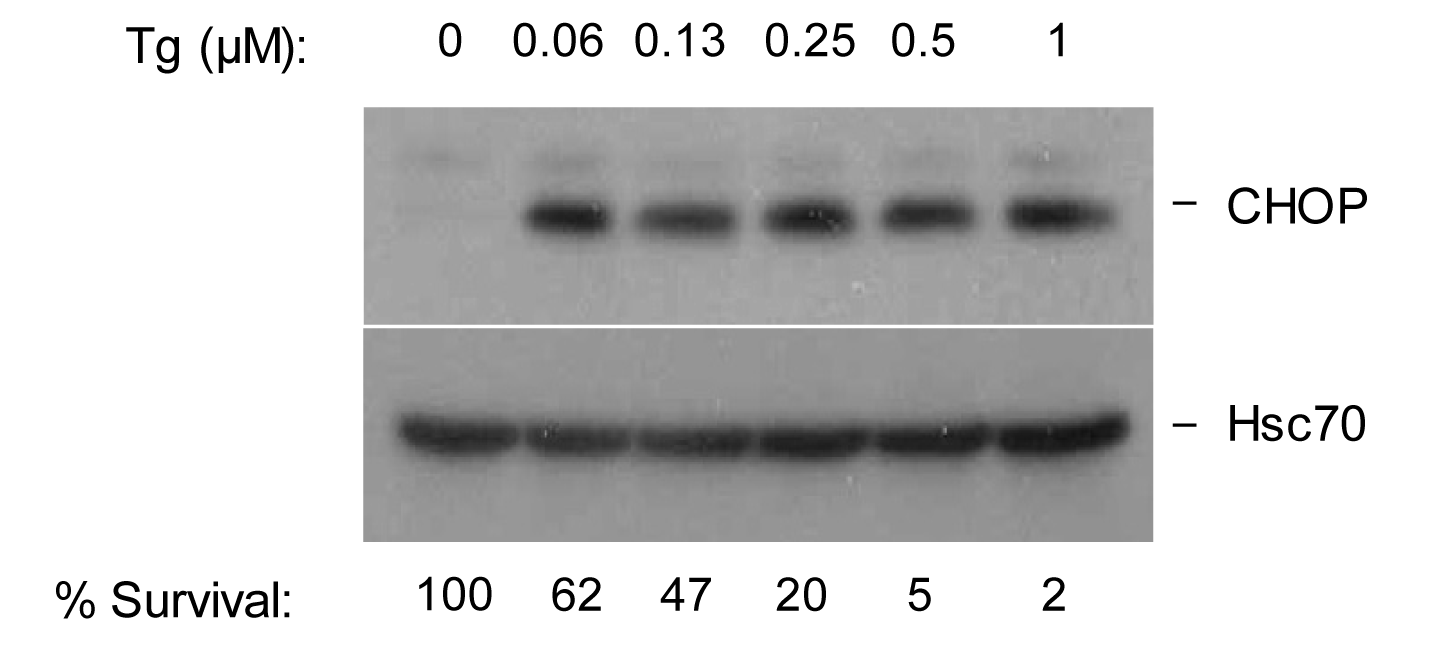

Supplement: Figure S2 — The non-doxycline treated SK-N-AS clone was treated with the indicated concentrations of thapsigargin for 6 hours. Cell lysates were prepared and analyzed by western blotting with the indicated antiserum. Hsc70 serves as a control for lysate loading. Cell viability was determined using the Pierce CellTiter assay as described in materials and methods and is indicated as % survival under each lane. (TIF) [file pone.0047931.s002.tif]

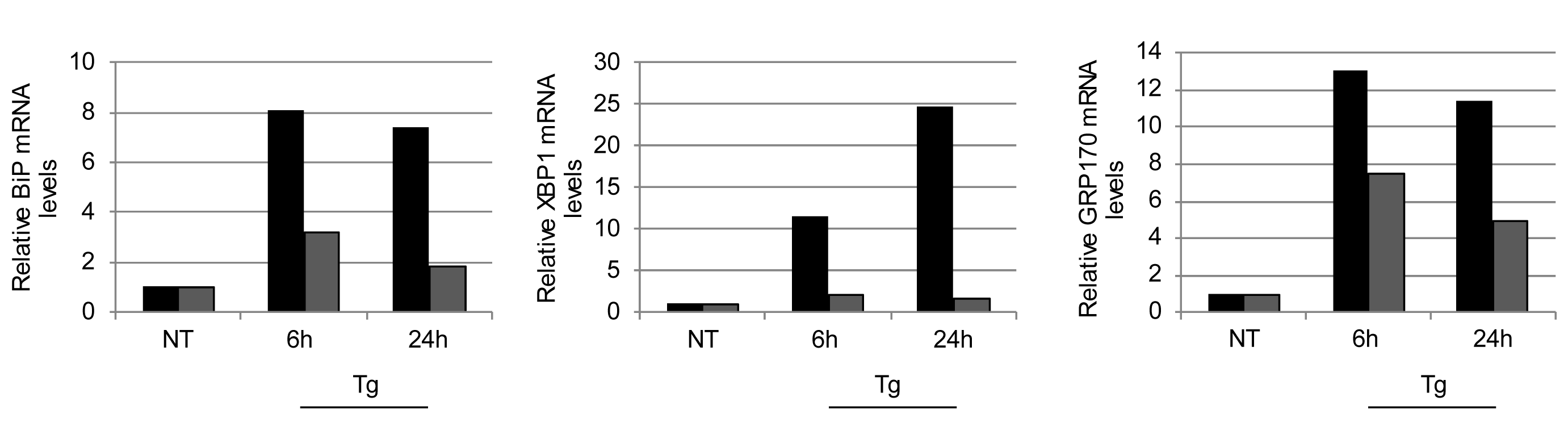

Supplement: Figure S3 — 293T cells were transiently transfected with either pCDNA3- empty vector (black bars) or pCGN-ATF6- dominant negative vector (grey bars). After 24 hours the cells were treated with thapsigargin (1 µM) for either 6 hours or 24 hours as indicated in the figure. Total RNA from the indicated samples was extracted and subjected to qRT-PCR to quantify BiP, XBP-1, and GRP170 mRNA levels. RNA levels were expressed relative to the control untreated samples transfected with each of the vectors, which was set to 1. (TIF) [file pone.0047931.s003.tif]
